# Intracranial Administration of P Gene siRNA Protects Mice from Lethal Chandipura Virus Encephalitis

**DOI:** 10.1371/journal.pone.0008615

**Published:** 2010-01-07

**Authors:** Satyendra Kumar, Vidya A. Arankalle

**Affiliations:** National Institute of Virology, Pune, India; Institut Pasteur, France

## Abstract

**Background:**

In parts of India, Chandipura Virus (CHPV) has emerged as an encephalitis causing pathogen in both epidemic and sporadic forms. This pediatric disease follows rapid course leading to 55–75% mortality. In the absence of specific treatment, effectiveness of RNA interference (RNAi) was evaluated.

**Methods and Findings:**

Efficacy of synthetic short interfering RNA (siRNA) or short hairpin RNA (shRNA) in protecting mice from CHPV infection was assessed. The target genes were P and M genes primarily because important role of the former in viral replication and lethal nature of the latter. Real time one step RT-PCR and plaque assay were used for the assessment of gene silencing. Using pAcGFP1N1-CHPV-P, we showed that P-2 siRNA was most efficient in reducing the expression of P gene *in-vitro*. Both quantitative assays documented 2logs reduction in the virus titer when P-2, M-5 or M-6 siRNAs were transfected 2hr post infection (PI). Use of these siRNAs in combination did not result in enhanced efficiency. P-2 siRNA was found to tolerate four mismatches in the center. As compared to five different shRNAs, P-2 siRNA was most effective in inhibiting CHPV replication. An extended survival was noted when mice infected intracranially with 100 LD_50_ CHPV were treated with cationic lipid complexed 5 µg P-2 siRNA simultaneously. Infection with 10LD_50_ and treatment with two doses of siRNA first, simultaneously and second 24 hr PI, resulted in 70% survival. Surviving mice showed 4logs less CHPV titers in brain without histopathological changes or antibody response. Gene expression profiles of P-2 siRNA treated mice showed no interferon response. First dose of siRNA at 2hr or 4hr PI with second dose at 24hr resulted in 40% and 20% survival respectively suggesting potential application in therapy.

**Conclusions:**

The results highlight therapeutic potential of siRNA in treating rapid and fatal Chandipura encephalitis.

## Introduction

Chandipura virus (CHPV), an emerging encephalitis-causing pathogen in India belongs to family *Rhabdoviridae* and genus vesiculovirus. Outbreaks of CHPV have been reported from the states of Andhra Pradesh [1], Gujarat [2] and Maharashtra [National Institute of Virology (NIV), unpublished data] with mortality varying from 55–75% in children. In an endemic area, sporadic disease has also been recorded [3]. CHPV has a negative sense RNA genome of approximately 11.0 Kb encoding 5 different proteins; nucleocapsid (N) protein, phosphoprotein (P) protein, matrix (M) protein, glycoprotein (G) protein and large (L) protein. The P protein has important functions in the virus life cycle, both in transcription and replication [4]

RNA interference (RNAi) is a post-transcriptional process triggered by the introduction of double-stranded RNA (dsRNA), which is subsequently cleaved by Dicer into 21–23 nt small interfering RNAs (siRNA). The siRNAs are then unwound by RISC (RNA induced silencing complex) in the presence of ATP. The activated RISC binds and degrades target mRNA guided by the single strand of the siRNA [5]–[12].

In recent years, RNAi has emerged as a powerful tool for gene silencing with a potential for therapeutic use in viral infections such as Human immunodeficiency virus (HIV) [13] Influenza [14], Respiratory syncytial virus (RSV) [15], SARS coronavirus [16] and Ebola virus [17].

Several studies have shown that the central nervous system (CNS) is also acquiescent to RNAi as in the case of brain cancer [18], Spinocerebellar ataxia [19]. Intracranial injection of siRNA has shown immense potential in suppressing encephalitis caused by two flaviviruses, JEV (Japanese encephalitis virus) and WNV (West Nile virus) [20]. Control of non-segmented negative-strand RNA virus replication in culture cells by siRNA has been well documented [21]. A recent study has shown inhibition of rabies virus replication by multiple artificial microRNAs (amiRNA) in Neuro 2a cells [22].

Considering the recent emergence of a rapidly progressing viral infection leading to encephalitis with high fatality proportion in children from India and the absence of any specific treatment, RNAi based interventions were explored to suppress CHPV infection in mice.

## Materials and Methods

### Cells and Virus used in study

Human embryonic Kidney (HEK)-293 cells were used for the siRNA studies and Vero E6 cells were used for the plaque assay. Chandipura Virus 034627, isolated during 2003 Andhra Pradesh epidemic [1] was used throughout the study.

### Cloning and expression of P gene of CHPV in pAcGFP1N1

P gene of CHPV was amplified by reverse transcriptase – polymerase chain reaction (RT-PCR) using PFloF1 (5′-TCTCGAGCTCAAGCTCCACCATGGAAGACTCTCAACTAT-3′) and PGFPR1 (5′-CCGCGGTACCGTCGAATTGAACTGGGGTTCAAG-3′) primers without stop codon and cloned into pAcGFP1N1 vector (named pAcGFP1N1-CHPV-P). HEK-293 cells were transfected with 2 µg each of pAcGFP1N1 and pAcGFP1N1-CHPV-P. Transfected cells were observed under florescent microscope periodically to check the expression of green fluorescent protein (GFP) fused CHPV P protein and confirmed by western blotting using anti-GFP antibodies (Invitrogen, Carlsbad, CA, USA).

### Design and synthesis of siRNA

**Figure 1:**
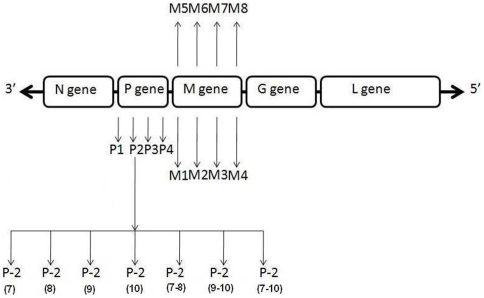
Schematic representation of gene arrangement in CHPV and siRNAs used. P and M genes were targeted for siRNA synthesis. The numbers were assigned arbitrarily and do not indicate location (refer table 1 for correct location of siRNA). Different siRNA used to determine tolerance for mismatches using P-2 siRNA number in parentheses represent the positions at which mismatches were incorporated (For sequence of P-2 siRNA mutants refer table 2).

All CHPV sequences present in the GenBank [23] and the sequences of the new isolates generated in the lab (NIV, unpublished data) were used to design the siRNA. All siRNAs were designed using HP OnGuard siRNA design. (Table 1 and Fig 1). Four and 8 siRNAs were synthesized for P gene and M gene respectively targeting cDNA (+) strand. Negative control siRNA (ncsiRNA) with no significant homology to any known mammalian gene was used as a non-silencing control in all RNAi experiments and purchased from qiagen (Catalog number 1027280) and negative control plasmid (NCP) was used in shRNA experiments (Ambion, USA).

(http://www1.qiagen.com/Products/GeneSilencing/SiRnaDuplexes/HPGuaranteed.aspx).

**Table 1:** Sequences of the siRNAs (target) located in P gene [CIN0327R (AY614726)] and M gene (AF128868).

Name	Sequences	Location
P-1	AGCAATTGATAAAGAATTCAT	1968–1988
P-2	ACCGAATCACCTGGCTCCAAA	1894–1914
P-3	CAGTTCAATTGAAATGATGCA	2173–2193
P-4	GAGAGGAATTCCCAGCTATTA	1428–1448
M-1	ATGGAATATTCTTACAAACAA	633–653
M-2	AAGCTTCAGTATAAATGTGTA	337–357
M-3	ATGGAACAGCTCGATGGATTA	201–221
M-4	CAACACATGGCTGATTTCTAA	801–821
M-5	CCCGGTCAATTTCCGAGACGA	723–743
M-6	TGCGATCAATCCATTCAGAGA	369–389
M-7	CAGTGGATACATTGGGAAGAA	435–455
M-8	TCCATGATGTTTGTCCCAGAA	598–618

**Table 2:** Mutant siRNAs used for determining tolerance of mismatches in the P-2 siRNA.

S. No	Name	Nucleotide	Target sequence (Change is shown in Bold)
1.	P-2 (7)	(A --**T** )	CGAATC**T**CCTGGCTCCAAA
2.	P-2 (8)	(C--**G** )	CGAATCA**G**CTGGCTCCAAA
3.	P-2 (9)	(C--**G** )	CGAATCAC**G**TGGCTCCAAA
4.	P-2 (10)	(T--**A** )	CGAATCACC**A**GGCTCCAAA
5.	P-2 (7–8)	(AC-**TG** )	CGAATC**TG**CTGGCTCCAAA
6.	P-2 (9–10)	(CT-**GA** )	CGAATCAC**GA**GGCTCCAAA
7.	P-2 (7–10)	(ACCT-**GTTC** )	CGAATC**GTTC**GGCTCCAAA

### Transfection of siRNA

For the transfection of siRNA, amaxa nucleofactor II device and supplemented kit V solution was used as suggested by the manufacturer. For transfection using HiPerfect reagent, HEK-293 cells were seeded 12–16 hr before transfection in a six-well plate (1×10^5^ per well). Lipid-siRNA complexes were prepared by incubating 100 pmol of siRNA with HiPerfect reagent (QIAGEN, Valencia, CA) in the appropriate volume as recommended by the manufacturer. To determine transfection efficiency, P-2 siRNA tagged with Alexa Fluor-647 was used (Qiagen, Valencia, CA).

### P gene siRNA validation with pAcGFP1N1-CHPV-P

Cells were transfected with P gene siRNA (P-1 to P-4) at a concentration of 100 pmol and 2 µg of target expressing plasmid pAcGFP1N1-CHPV-P. 24 hr post transfection, cells were harvested and flow cytometry and real time one step RT-PCR were carried out. For real time one step RT-PCR, RNA was isolated using RNAeasy minikit (QIAGEN, Valencia, CA) as described by the manufacturer. To remove plasmid 80U RNase-free DNase™ (Promega, Madison, USA) enzyme was used. 500 ng of total RNA was used for real time one step RT-PCR. Each real time one step RT-PCR assay had at least two no amplification controls (NAC) in which RT enzyme was substituted by DNase-RNase free water to check for the DNA contamination.

### P gene and M gene validation with CHPV

HEK-293 cells were infected with 360 plaque forming units (PFU) of CHPV. Cells were transfected with 100 pmol of different siRNAs at 2 hr PI. Cell supernatants were harvested 24 hr post transfection, RNA isolated using QIAmp viral RNA minikit (QIAGEN, Valencia, CA) and real time one step RT-PCR was carried out as described earlier [24].

### Effect of combination of siRNA on the replication of CHPV

To determine the effect of combination of siRNAs on the replication of CHPV, a concentration of 100 pmol of all the siRNAs was used alone or in different combinations.

### Real time one step RT-PCR, flowcytometry and plaque assay to assess gene silencing

Total RNA from cells transfected with pAcGFP1N1-CHPV-P, mouse brain samples and viral RNA from supernatant of CHPV infected cells were subjected to real time one step RT-PCR as described earlier [24].

For flowcytometry acquisitions were done using FACScalibur (BD Bioscience) and analyses were performed using cell quest software (BD Bioscience).

For plaque assay Vero cells were grown to near confluence in 24-well tissue culture plate. The cells were adsorbed with 10-fold serially diluted supernatant containing CHPV at 37°C for 60 min. Following adsorption, the cells were washed with PBS and fed with 1 ml of 2X MEM (Sigma, USA) along with 1.25% (w/v) carboxymethyl cellulose (Sigma, USA). The cells were incubated for 2 days and observed for plaques under an inverted microscope and stained with crystal violet (0.1%) after fixing in 10% (v/v) formal saline. The plaques were counted and the titer of the virus was calculated as PFU/ml [25].

### Tolerance for mismatches

To investigate the tolerance of RNAi system for mismatches in the siRNA relative to target mRNA, different siRNAs with mismatches at specific positions as shown in Table 2 and Fig 1 were designed.

### Comparison of P-2 siRNA and P-2 short hairpin RNA (shRNA) efficiency

To deliver the siRNA in shRNA format and to compare efficiency of the siRNA and shRNA, the sequence of the P-2 siRNA was subjected to the link www.ambion.com/techlib/misc/psilencer_converter.html. DNA oligos were synthesized and named as pH1-P2 or pCMV-P2 depending upon the plasmid promoter used for the expression of shRNA. Following a report documenting hypersusceptibility of Dicer-1-deficient mice to VSV infection as a result of impaired miR-24 and miR-93 expression [26], we examined the contribution of miR-93 in the replication of CHPV. Use of bioinformatics tools, RNA22 (http://cbcsrv.watson.ibm.com/rna22_targets.html) and RNAhybrid (http://bibiserv.techfak.uni-bielefeld.de/rnahybrid/submission.html) revealed that CHPV P gene has two potential binding site for hsa-miR-93. After confirmation, the role of miR-93 in the CHPV replication was assessed by using the stem and loop of miR-93 in different combinations using pSilencer 2.0 H1 neo vector (Ambion, Applied Biosystems International, Foster City, CA) and named pH1-mir-93 (hsa-miR-93 precursor sequence), pH1-miR-93-P-2 (stem of miR-93 was replaced with P-2 siRNA) and pH1-mir-93-P-2-30 (stem and loop of miR-93 was replaced with P-2 siRNA and miR-30 respectively) was used. All the oligos were synthesized by IDT, USA (Table 3). Sense and antisense oligos were annealed and ligated to either pSilencer 3.1 H1 neo or pSilencer 4.1 CMV neo vectors (Ambion, Applied Biosystems International, Foster City, CA) as recommended by the manufacturer. All the clones were screened by restriction digestion and confirmed by sequencing. Endofree maxi preps were made and quantitated. Different shRNA expressing plasmids (5 µg) were co-transfected individually with plasmid pAcGFP1N1-CHPV-P (500 ng). 24 hr post transfection, cells were harvested, RNA isolated and real time one step RT-PCR and FACS analysis were carried out. Different shRNA expressing plasmids were individually transfected 24 hr at post transfection, cells were infected with 36 PFU/ml of CHPV. At 12 and 24 hr post PI, supernatants were assayed for the presence of the CHPV by plaque assay and real time one step RT-PCR.

**Table 3:** Sequences of the shRNA oligos used.

S No	Name of the oligo	Sequences of the oligo
1	pH1-P2 sense	GATCCGCGAATCACCTGGCTCCAAATTCAAGAGATTTGGAGCCAGGTGATTCGGTTTTTTTGGAAA
2	pH1-P2 antisense	AGCTTTTCCAAAAAAACCGAATCACCTGGCTCCAAATCTCTTGAGTTTGGAGCCAGGTGATTCGCG
3	pCMV-P2 sense	GATCCCGAATCACCTGGCTCCAAATTCAAGAGATTTGGAGCCAGGTGATTCGGTA
4	pCMV-P2 antisense	AGCTTACCGAATCACCTGGCTCCAAATCTCTTGAATTTGGAGCCAGGTGATTCGG
5	pH1-miR-93 sense	GATCCGCTGGGGGCTCCAAAGTGCTGTTCGTGCAGGTAGTGTGATTACCCAACCTACTGCTGAGCTAGCACTTCCCGAGCCCCGGTTTTTGGAAA
6	pH1-miR-93 antisense	AGCTTTTCCAAAAAACCGGGGGCTCGGGAAGTGCTAGCTCAGCAGTAGGTTGGGTAATCACACTACCTGCACGAACAGCACTTTGGAGCCCCCAGCG
7	pH1-miR-93-P-2 sense	GATCCGCTGGGGGCTCCAGCGAATCACCTGGCTCCAAATGTGATTACCCAACTTTGGAGCCAGGTGATTCGCCCCGAGCCCCCGGTTTTTTGGAAA
8	pH1-miR-93-P-2 antisense	AGCTTTTCCAAAAAACCGGGGGCTCGGGGCGAATCACCTGGCTCCAAACTTGGGTAATCACATTTGGAGCCAGGTGATTCGCTGGAGCCCCCAGCG
9	pH1-miR-93-P-2-30 sense	GATCCGCTGGGGGCTCCAGCGAATCACCTGGCTCCAAATGTGGTGAAGCCACAGATGCAACTTTGGAGCCAGGTGATTCGCCCCGAGCCCCCGGTTTTTTGGAAA
10	H1-miR-93-P-2-30 antisense	AGCTTTTCCAAAAAACCGGGGGCTCGGGGCGAATCACCTGGCTCCAAAGTTGCATCTGTGGCTTCACCACATTTGGAGCCAGGTGATTCGCTGGAGCCCCAGCC

### Mouse infection

BALB/c mice aged 4–6 wks were used for all *in vivo* experiments. These experiments were done in a biosafety level 2 animal facility at the NIV and were approved by the Institutional Animal Ethics Committee (IAEC) for animal experimentations. Mice were inoculated intracranially (ic) with CHPV. For experiments using siRNA, siRNAs were complexed with HiPerfect™ (QIAGEN, Valencia CA) according to the manufacturer's instructions and were administered at the same site once simultaneously or at different intervals.

### Mouse tissue preparation for real time one step RT-PCR, gene expression and histopathology

Mice (n = 3) were euthanized and brains/spleens were removed and used in various experiments. For histopathology, the brain samples were fixed in 10% neutral buffered formalin, embedded in paraffin and 6-µm vertical sections were stained with hematoxylin and eosin. For the quantitative assessment of CHPV replication in mice brain using real time one step RT-PCR, RNA isolated using Ribopure RNA isolation Kit (Ambion, USA) according to manufacturer's instructions and 500 ng of total RNA was used per reaction [24].

Gene expression analysis using TaqMan Low Density Array (TLDA), single-cell suspension of spleens were prepared by gently teasing in RPMI medium and RNA isolated as above. RNA from spleens of the mice (n = 3) from same group were pooled and samples were run in duplicate on 7900-HT fast real time PCR machine (Applied Biosystems International, Foster City, CA). Data analysis was done using RQ software, with respect to control (uninfected/untreated) and HPRT was used as endogenous control.

### Statistical analysis

Statistical analyses were done using XLSTAT 2009 program in Excel 2007 software. Three independent experiments were performed in triplicates. Results are expressed as means ± SD. Data were analyzed by one way ANOVA followed by Dunnett's test. For *in vivo* experiments, mice survivals were compared using Log-rank (Mental Cox) and Gehan-Breslow-Wilcoxon tests and GraphPad Prism software 5.0 evaluation version.

## Results

### Expression of P gene fused with GFP

For the initial rapid and efficient assessment of gene silencing, P gene of CHPV was expressed with GFP as fusion protein. Fluorescent microscopy and Western blotting confirmed expression of P protein fused with GFP. The molecular weight of the fusion protein was approximately ∼61.0 Kda. The molecular weight of the GFP protein is 29.0 Kda and the molecular weight of bacterial expressed P protein is 32.5 Kda [27]. Confocal microscopy of HEK-293 cells transfected with pAcGFP1N1 and pAcGFP1N1-CHPV-P showed cytoplasmic localization of fusion protein

### P gene siRNA validation with pAcGFP1N1-CHPV-P and CHPV

**Figure 2:**
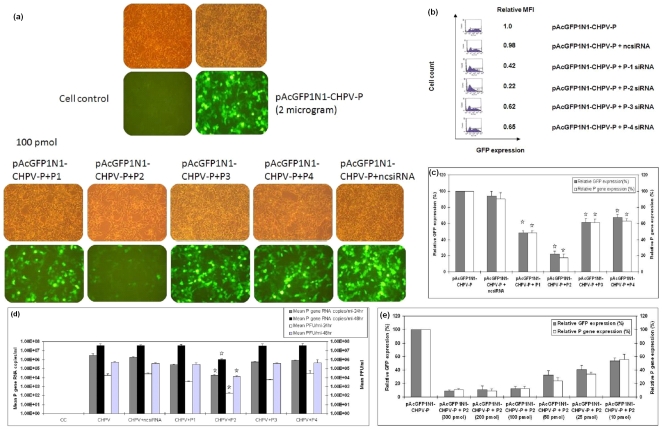
Validation of P gene siRNA using pAcGFP1N1-CHPV-P. (**a**) Fluorescent microscopic images of HEK-293 cells transfected with different P gene siRNAs and plasmid pAcGFP1N1-CHPV-P. (**b**) P gene siRNAs validated by flowcytometry at the protein level (**c**) Reduced expression of P gene mRNA by real time one step RT-PCR. (**d**) P gene validation with CHPV at different time points. (**e**) Effect of different doses of P-2 siRNA on the expression of P gene as determined by flowcytometry and real time one step RT-PCR. Result are expressed as means ± SD. * indicates significantly different from control at p <0.05 by Dunnett's test.

At 24 hr post transfection, cells transfected with both P gene siRNAs (P-1 to P-4) and target expressing plasmid pAcGFP1N1-CHPV-P showed that P-2 siRNA could reduce the GFP expression and P gene expression approximately by 80% (Fig 2a-c c) (p<0.05), whereas P-1, P-3 and P-4 were not very effective. With respect to CHPV, efficacy of P-2 siRNA in reducing replication was 2logs at 24 hr and 1log at 48hr (p<0.05) as evidenced by plaque assay and real time one step RT-PCR (Fig 2d). A dose response experiment using 10–300 pmol of P-2 siRNA and pAcGFP1N1-CHPV-P showed 100 pmol as optimum concentration (Fig 2e).

### Validation of M gene siRNAs with virus and comparison with P-2 siRNA

**Figure 3:**
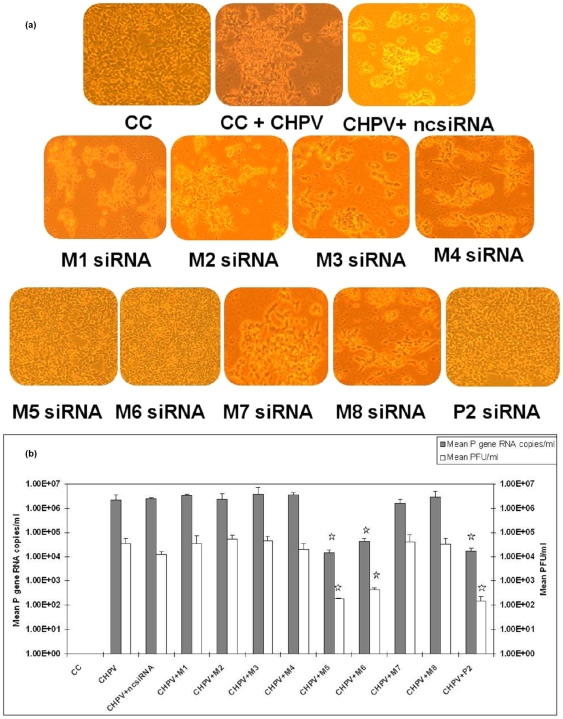
Validation of P-2 and M gene siRNA with CHPV. (**a**) Inhibition of CHPV induced cytopathic effects by siRNAs as observed by phase-contrast microscopy (10X). (**b**) Effect of different siRNAs on the replication of CHPV as determined by real time one step RT-PCR and plaque assay.Results are expressed as means ± SD. * indicates significantly different from control at p<0.05 by Dunnett's test.

M gene siRNAs (M1-M8) were tested with the virus and efficiencies were compared with P-2 siRNA. The results showed P-2, M-5 and M-6 to be equally potent in inhibiting CHPV induced cytopathic effects as observed by phase-contrast microscopy (10X magnification) (Fig 3a). Assessment of virus replication by plaque assay and real time one step RT-PCR showed inhibition of CHPV by 2logs and thus confirmed efficacy of all the three siRNAs (p<0.05) (Fig 3b).

### Effect of combination of siRNAs on the replication of CHPV

**Figure 4:**
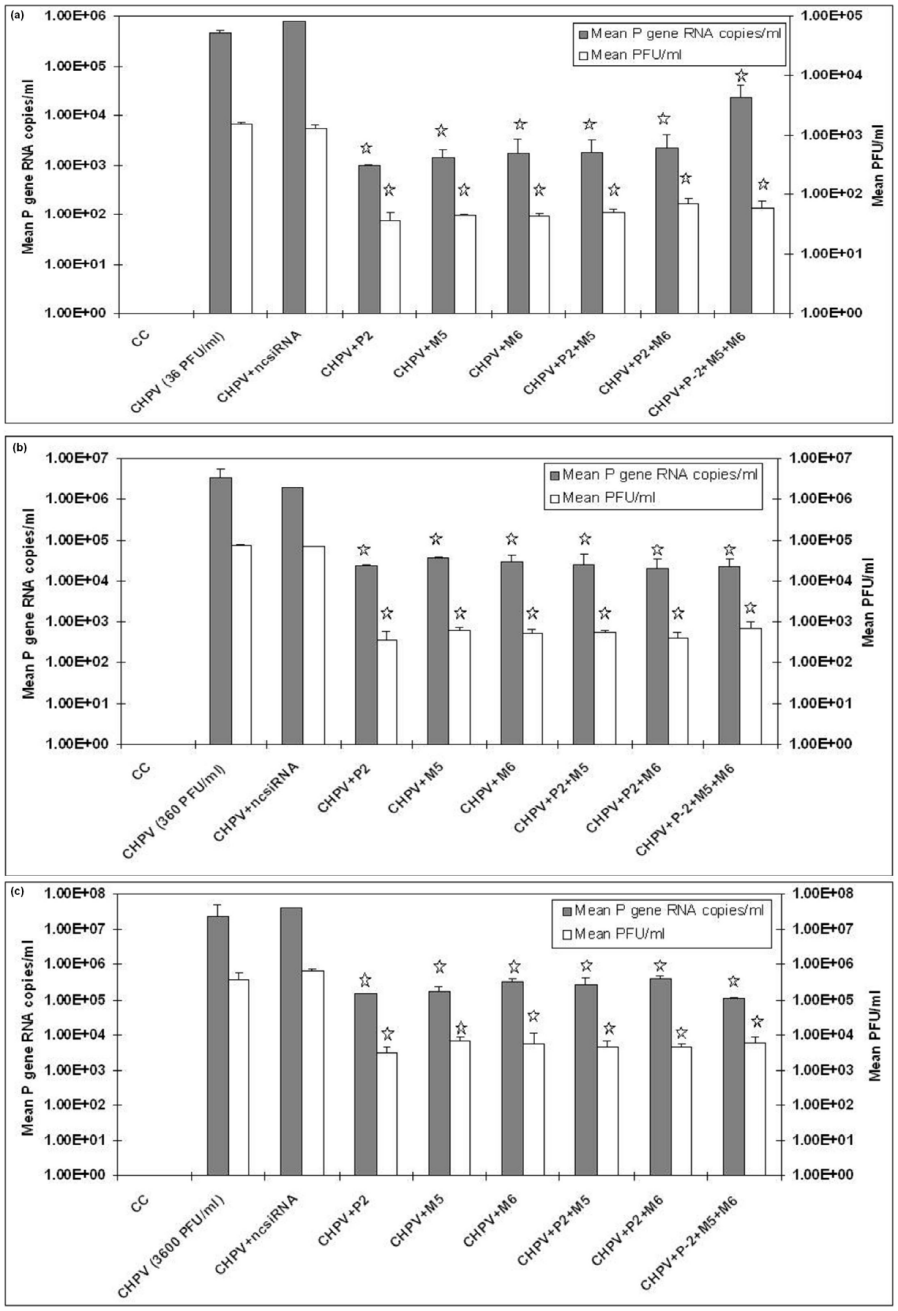
Effect of P-2, M-5 and M-6 siRNAs on the replication of CHPV. (**a**) The virus inoculum was 36 PFU/ml, (**b**) 360 PFU/ml and (**c**) 3600 PFU/ml. Results are expressed as means ± SD. * indicates significantly different from control at p<0.05 by Dunnett's test.

Initially, we tried combinations of P-2, M-5 and M-6 siRNAs at different concentrations (10, 25, 50, 100 and 200pmol of each siRNA) against 36, 18 and 3.6 PFU/ml. Cells could not be infected below 18PFU and no inhibition was observed at 10 and 25pmol concentrations of siRNAs. At 50 pmol, 1log and at 100 and 200pmol optimum reduction (2logs) was achieved. Figure 4 depicts efficiency of P-2, M-5 and M-6 siRNAs when used alone or in different combinations (P-2+M-5; P-2+M-6 and P-2+M-5+M6) at 100pmol of each siRNA. In each case,. 2logs reduction in viral titer as compared to controls was recorded (p<0.05). Infection with CHPV at as low as 36 PFU/ml and as high as 3600 PFU/ml did not alter the efficiency of the siRNA the reduction being 2logs in all cases (Fig 4a-4c).

### Tolerance for mismatches

**Figure 5:**
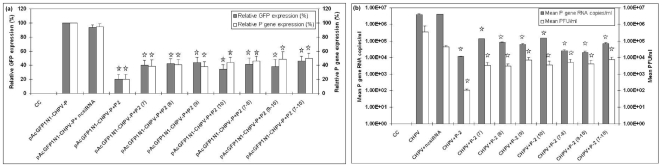
Tolerance of RNAi for mismatches in the P-2 siRNA relative to target mRNA. Tested *in vitro* by using (**a**) pAcGFP1N1-CHPV-P plasmid and (**b**) CHPV. Results are expressed as means ± SD. * indicates significantly different from control at p<0.05 by Dunnett's test.

P-2 siRNA could tolerate mismatches at single nucleotide (positions 7, 8, 9 and 10) two adjacent mutations (at 7–8 and 9–10) or four consecutive positions (at 7–10) as judged by both pAcGFP1N1-CHPV-P and CHPV. Results showed at least 50% P gene silencing (Fig 5a) (p<0.05) and 1log difference in the titter of CHPV in mutant siRNA treated and control cells (Fig 5b) (p<0.05).

### Short hairpin RNA (shRNA) validation with pAcGPF1N1-CHPV-P plasmid and with CHPV

**Figure 6:**
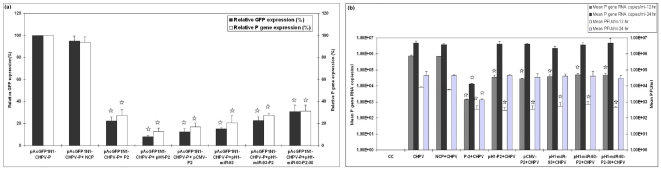
Validation and comparison of efficiency of P-2 siRNA with different P-2 shRNA. (**a**) Validation of different shRNA expressing plasmids with pAcGFP1N1-CHPV-P by using FACS and real time one step RT-PCR and (**b**) Efficiency of different shRNA expressing plasmids in modulating CHPV replication at 12hr and at 24hr determined by real time one step RT-PCR and plaque assay. Results are expressed as means ± SD. * indicates significantly different from control at p<0.05 by Dunnett's test.

Flowcytometery and real time one step RT-PCR documented that P-2 shRNA expressed either from Pol III (pH1-P2) or Pol II promoter (pCMV-P2) was as efficient as P-2 siRNA, when the validation was done with pAcGFP1N1-CHPV-P. The reduction was approximately 70% (Fig 6a) (p<0.05).

The silencing effect lasted till 12hr (Fig 6b) (p<0.05) and was lost at 24hr when tested with CHPV. At 24hr there was no difference in the treated and control cells. P-2 siRNA was used as a positive control and showed activity similar to experiments conducted earlier.

### Effect of intracranial administration of P-2 siRNA on CHPV infection in mice

Transfection efficiencies of Alexa fluor-647nm-labeled P-2 siRNA either with nucleofaction or Hiperfect reagent in HEK-293 cells were 83 and 62% respectively, as measured by flowcytometry. When the cells infected with 360 pfu/ml CHPV were treated with P-2 siRNA, Hiperfect was less effective than nucleofaction (2logs reduction at 12hr for both and 1log less at 24hr). For all in-vivo experiments Hiperfect reagent was used for delivery of P-2 siRNA. As adult mice are not susceptible to CHPV by peripheral route, intracranial route was used for infection with the virus.

**Figure 7:**
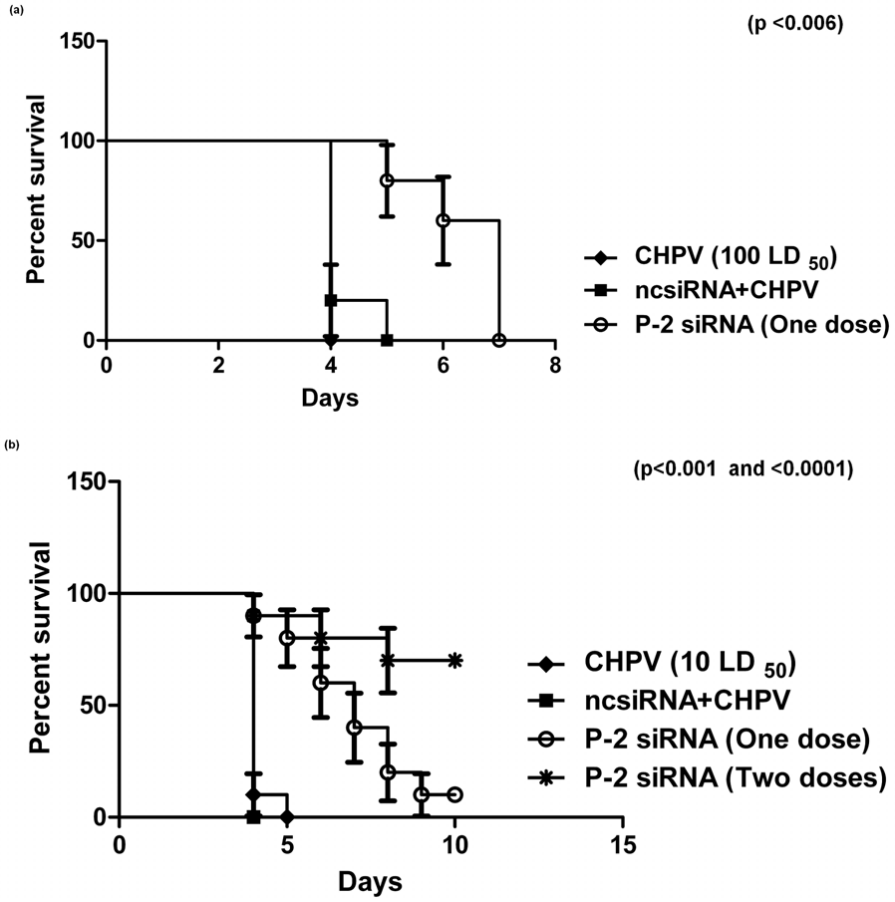
Kaplan Meier Survival curve showing effect of different doses of virus and siRNA. (**a**) 100LD_50_ CHPV and single dose of P-2 siRNA was administered (n = 5) (p<0.006). (**b**) 10 LD_50_ of CHPV and single or two doses (n = 10 for both) of P-2 siRNA (p<0.0001).

For the first experiment, 100LD_50_ CHPV was used. Simultaneous treatment either with P-2 siRNA (5 µg) or ncsiRNA (5 µg) complexed with Hiperfect at the same site led to survival of 5–7 days in former and 4–5 days in latter groups (Fig 7a) (p<0.006).

**Figure 8:**
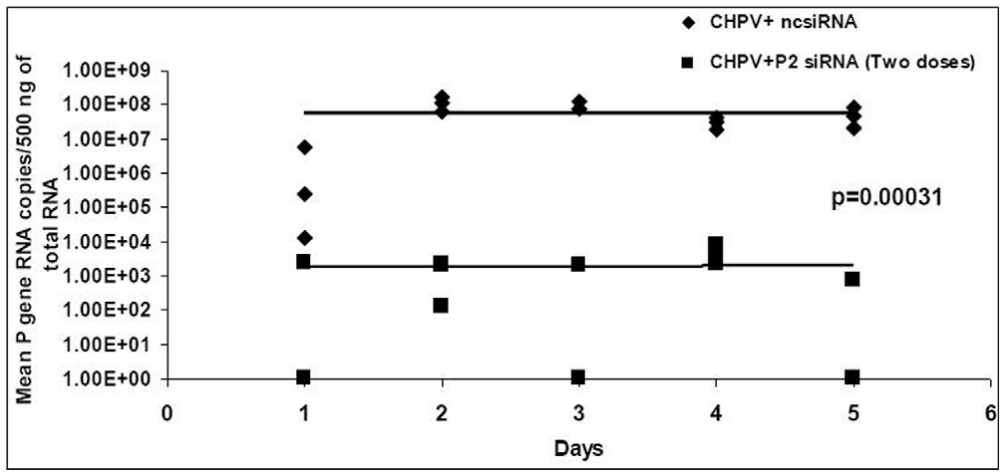
Reduction in the levels of CHPV replication in mice brain. Each day, brain homogenates from 3 mice were taken for RNA isolation and used for real time RT-PCR. Each symbol represents individual mouse. Data are shown only till all mice in control group died.

In another experiment, the virus dose was reduced to 10 LD_50._ Mice received one (simultaneous) or two doses (0 and 24hr) of 5 ug P-2 siRNA. In the first group, the animals showed extended survival by 48 hr (∼60% mice) followed by survival of one mouse (10%, (p<0.001). Importantly, ∼70% survival was recorded in mice receiving two doses of P-2 siRNA (p<0.0001) (Fig 7b). In accordance with the extended survival, the viral titres in the brain tissue from mice treated with two doses of P-2 siRNA were reduced by 4logs as compared to the control group (p<0.00031) (Fig 8).

**Figure 9:**
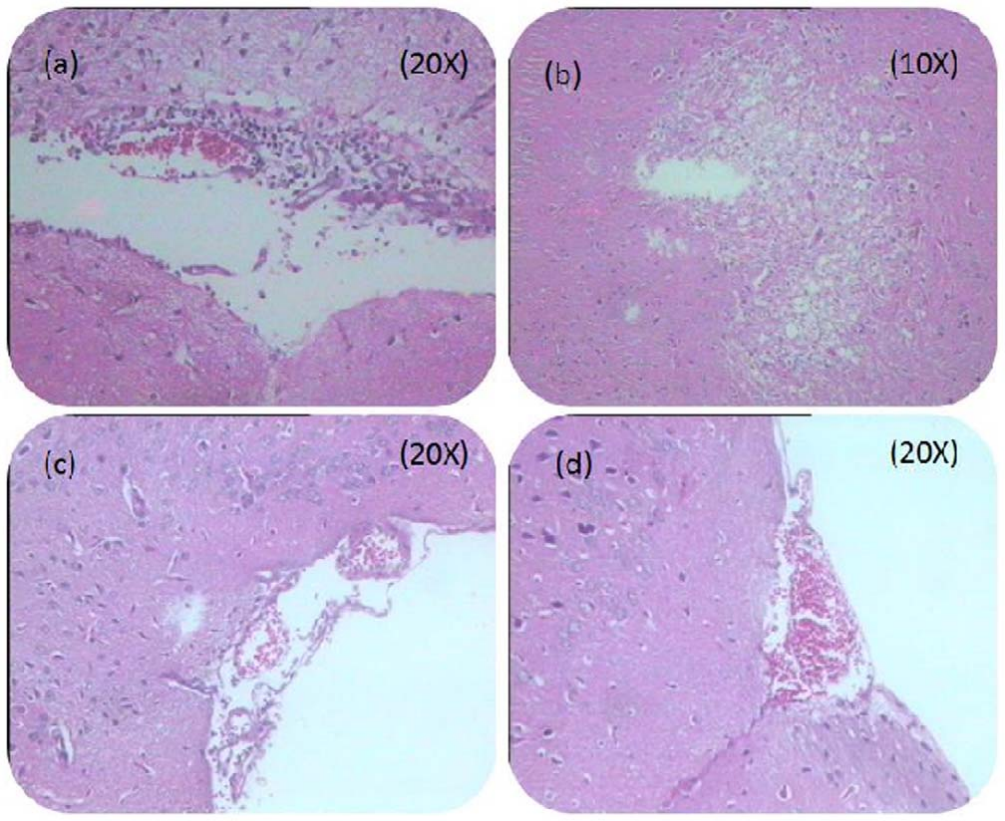
Histopathology of mice brain treated with P-2 siRNA. Hematoxylin and eosin-stained vertical brain sections from control mice treated with ncsiRNA and infected with CHPV (**a, b**) and P-2 siRNA treated mice on 5^th^ day PI (**c, d**) at indicated magnifications.

Histopathology of the brains of ncsiRNA treated mice showed typical features of viral encephalitis, such as meningeal vessels with perivascular cuffing of lymphocytes (Fig 9a) and focus of gliosis & demyelination in cortex (Fig 9b). Absence of brain inflammation or neuropathology was obvious in P-2 siRNA-treated mice (Fig 9c and 9d). Both mice groups were sacrificed on 5^th^ day post-CHPV inoculation.

**Figure 10:**
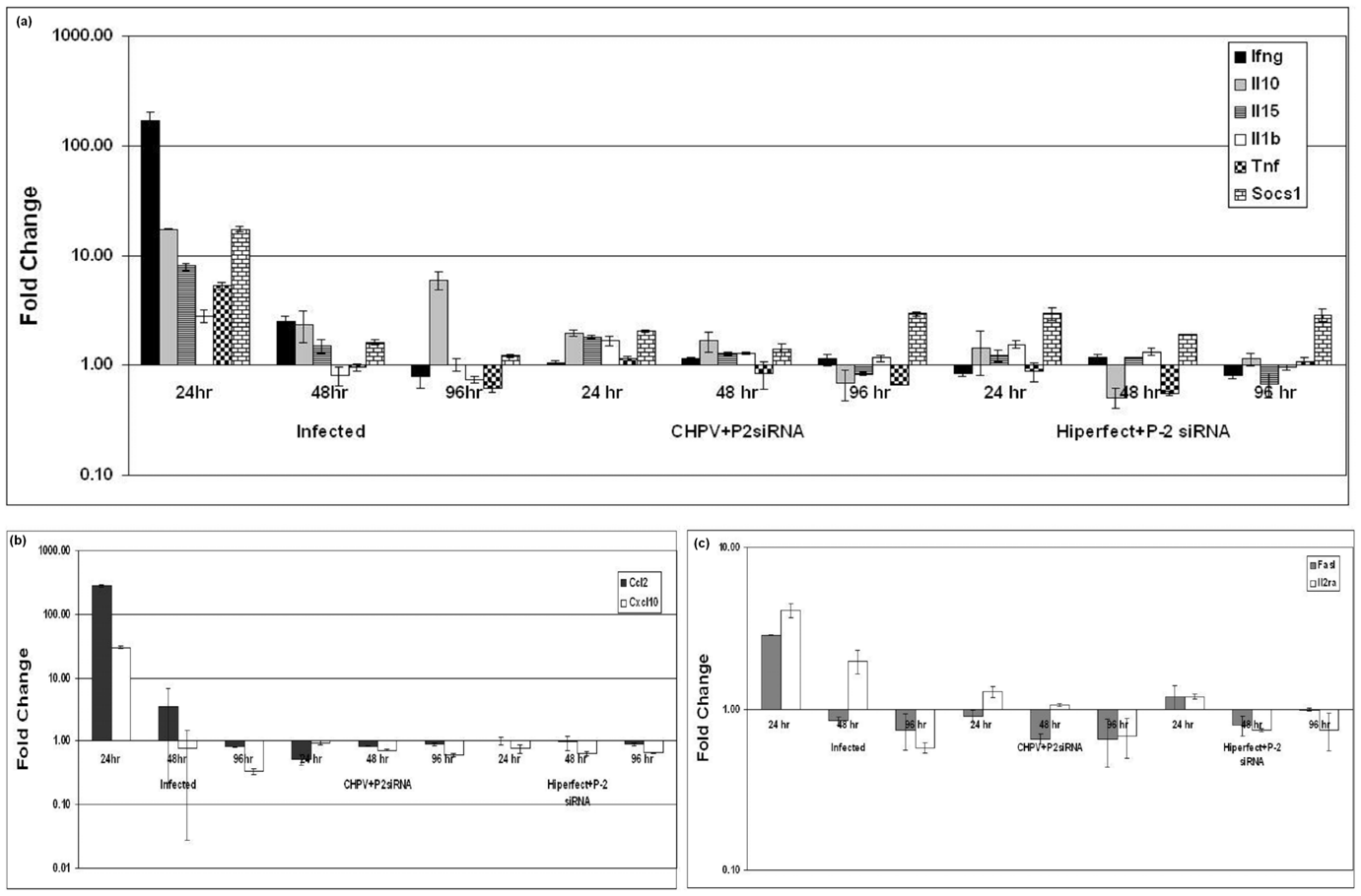
Gene expression profiles of siRNA treated mice using TLDA. (**a**) cytokines genes, (**b**) chemokine genes and (**c**) cell surface molecules in spleen of mice from 3 groups- (1) CHPV infected control (2) CHPV infected and P-2 siRNA treated and (3) Hiperfect + P-2 siRNA inoculated control. Results are expressed as means ± SD.

In order to rule out the induction of non-specific interferon responses by P-2 siRNA, TLDA was performed. Gene expression changes ≥2.5 fold were considered significant. Expression of IFN-γ (>100-fold), IL-10 (∼17fold), IL-1β (2.8 fold), IL-15 (∼8-fold), SOCS-1(∼17-fold) and TNF (∼5-fold) cytokines genes were upregulated at 24hr PI. A significant reduction in the expression of IFN-γ (∼2.5-fold) was noted at 48 and 96hr PI. IL-10 (∼2.3-fold) decreased at 48hr but increased at 96hr (6-fold) while SOCS-1 decreased at all the time points (Fig 10a).

Expression of the chemokines genes (Ccl-2, >200-fold; csf2, >10-fold and Ccxl-10, ∼30-fold) and surface receptor molecules (FasL, >2.5 fold and IL-2R, 4-fold) were upregulated at the initial phase of infection, but decreased at later time points (Fig 10b, c). None of the above changes observed in CHPV infected mice were recorded in mice treated with P-2 siRNA or control mice receiving P-2 siRNA complexed with Hiperfect. These data clearly show that the protection offered by P-2 siRNA is specific and does not reflect non-specific anti-viral response of IFN.

Anti-CHP-IgG antibodies were absent on days 7, 14 and 21 post-infection in P-2 siRNA treated, surviving mice.

**Figure 11:**
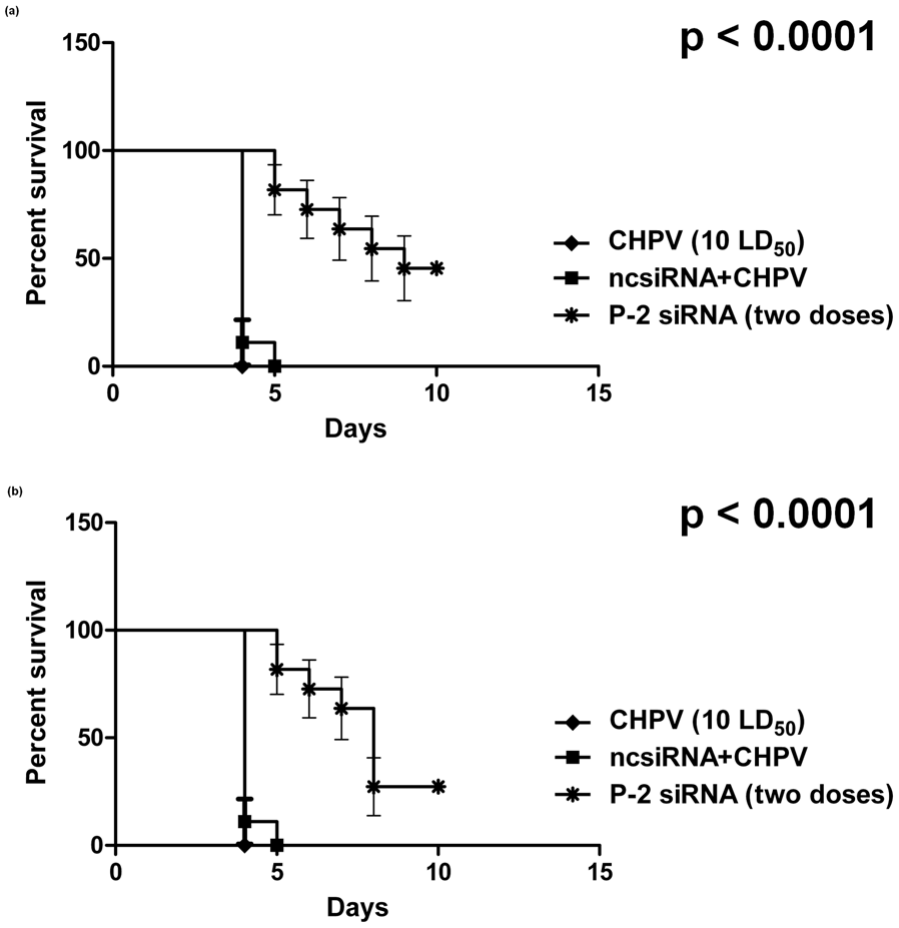
Kaplan Meier Survival curve showing efficiency of P-2 siRNA in clearing established CHPV replication. When administered (**a**) 2hr and (p<0.0001) (**b**) 4hr post virus inoculation (p<0.0001). In both cases the second siRNA dose was given at 24hr post infection.

Considering therapeutic importance, ability of P-2 siRNA to clear established CHPV infection was also evaluated. Two doses of siRNA (5 µg each 24hr apart) were found to give 40% and 20% protection respectively when the treatment was given 2hr and 4hr post-infection (p<0.0001) (Fig 11a, b).

## Discussion

Following the recognition of emergence of CHPV as an emerging pathogen causing rapid and high mortality in children in India, being the national institute and considering restricted spread of the virus, we undertook two approaches as probable strategies to combat the infection, development of a prophylactic vaccine [28] and siRNA administration as a probable therapeutic agent (present study). We were able to identify a potent siRNA that could protect mice from the lethal encephalitis even when the virus was directly introduced in the target organ, the brain.

The first step was selection of the viral gene(s) to be targeted by RNAi. We selected P and M genes as (1) P protein is indispensable for the activity of L protein which functions as RNA dependent RNA polymerase (RdRp) and (2) M protein is most lethal protein encoded by rhabdoviruses on account of induction of cytopathic effects and apoptosis in the cells [29].

Initially, a total of 12 siRNAs (4 for P gene and 8 for M gene) were screened *in-vitro*. Co-transfection of pAcGFP1N1-CHPV-P plasmid and all the P gene siRNAs identified P-2 siRNA as the most potent in perturbing P gene expression (Fig 2). M gene siRNAs were directly validated with the virus. P-2, M-5 and M-6 siRNAs were equally efficient in inhibiting CHPV induced cytopathic effects (Fig 3). Replication inhibition in siRNA treated cells was also confirmed by 2logs reduction in the virus titer as evidenced by both plaque assay and real time one step RT- PCR (Fig 3b). Unfortunately, use of combinations of effective siRNAs at different concentrations did not yield additive benefit. A possible explanation could be that cells have a limited capacity to assemble the RNA-induced silencing complex (RISC) and siRNA's compete for the available RISC pool present in the cells [30]. Similar observations have been reported for two respiratory viruses, respiratory syncytial virus and parainfluenza virus [15], [31].

To assess the specificity, inhibition of CHPV replication in P-2 and M-5/M-6 siRNA treated cells was assessed by G gene and P gene-based real time PCRs respectively (data not shown). In both cases, 2logs reduction was recorded. This may reflect the possibility of these siRNAs not only targeting their cognate mRNA but also the positive (+) strand pre-genomic RNA thus affecting overall replication of the virus.

So far, P gene / full genomes of CHPV isolated during 1965, 2003, 2004 and 2007 have been sequenced [23, our unpublished data]. Percent nucleotide identity (PNI) for P gene varied from 99.7 to 95.8%. Thus the virus has not changed appreciably over a period of 42 years. Despite the stable nature of the virus, tolerance of mismatches in the P-2 siRNA was examined as this molecule was to be evaluated in *in-vivo* experiments. Though mismatches in the center of the siRNA duplex are most critical abolishing target cleavage [32], [33], introduction of different mismatches did show inhibition of the virus, at reduced rate (Fig 5). Thus, even if the virus mutates, this siRNA will still be effective.

Considering several advantages offered by shRNA such as requirement for low doses, long-term downregulation of the target gene and an economic alternative to the siRNA, we also tried the shRNA format. Though efficient silencing was observed at 12hr, the efficacy was lost at 24hr (Fig 6b). This could possibly be because of apoptosis and subsequent inhibition of host transcription induced by M protein for both CHPV [34] and VSV [35] infections. Similar to our findings, abrogation of DNA based RNAi was found in apoptotic cells due to caspace-3 mediated inactivation of Dicer-1 without affecting RISC dependent part of RNAi [36]. Therefore, siRNA seems to be a better approach for regulating CHPV replication especially because it is an acute infection with rapid course and long-term treatment is not needed. However, in a recent study, efficacy of shRNA was effectively shown for the treatment of JEV encephalitis in mice by using lentiviral vector. 100% survival was recorded when mice were injected IC with pseudotyped RV-G lentiviral vector expressing shRNA specific for JEV and challenged half an hour later with 4 LD_50_ of JEV at the same site [20].

With the encouraging *in-vitro* results, we attempted to evaluate P-2 siRNA in a murine model. Availability and selection of suitable delivery system was the key question. Although successful use of naked siRNA targeting the pain-related cation channel P2X3 to treat chronic neuropathic pain in a rat model is reported [37], poor uptake by brain parenchymal cells was shown in other studies [38], [39]. Recently, a cationic lipid formulation, i-Fect (Neuromics) was found to deliver siRNA into neuronal cells without toxicity and could protect mice against viral encephalitis caused by flaviviruses [20]. We used P-2 siRNA complexed with a cationic lipid (Hiperfect) for delivery into brain.

Simultaneous administration of 5 µg P-2 siRNA and 100 or 10LD_50_ virus resulted in 48 and 96 hr delay respectively in the appearance of symptoms and death (Fig 7a and b). One mouse survived with the lower dose of the virus. Importantly, when two doses 5 µg P-2 siRNA were administered 24 hr apart, 70% mice survived. In this experiment, the first dose of siRNA was given simultaneously with the virus. To examine the degree of protection offered by siRNA when administered after the virus inoculation, a different two dose schedule was tried. i.e., 2 or 4 hr and 24hr-post-virus inoculation (Fig 11a and 11b). Survival was reduced to 40% and 20% respectively. Considering the rapidity of CHPV replication, these results are significant. If we could introduce large amount of siRNA post-infection, survival of mice seems likely. Due to the limitation of the volume, this could not be tried. It is pertinent to note here that for JE virus, a single injection containing 40ug of siRNA with JetSI/DOPE led to complete protection [20]. We need to try different liposome formulations encapsulating optimum concentration of P-2 siRNA for achieving near 100% survival when administered after infection with the virus at variable intervals.

For the siRNA dosing yielding optimum protection (2 doses, 24hr apart) additional parameters were evaluated. Daily monitoring of CHPV titers in the brain by real time one step RT-PCR (Fig 8) clearly showed 4logs reduction in mice treated with P-2 siRNA when compared to ncsiRNA treated controls. Histopathological analysis demonstrated that P-2 siRNA treated mice brains did not exhibit common features of viral encephalitis seen exclusively in the control group (Fig 9). In order to prove that the protection offered was indeed due to siRNA, we screened sera of survived mice at 7, 14 and 21 days after infection for the presence of anti-CHP-IgG antibodies. Absence of such antibodies demonstrated limited / no viral replication leading to no development of humoral response (Data not shown).

To generate additional information on the immune response, gene expression profiles were studied. For this, we used spleen and not brain primarily because our aim was not to dissect out effect of CHPV infection on the regulation of these genes in mice, but limited to compare the expression levels of immune response genes in among the siRNA treated and control mice receiving the same dose of the virus. In this regard a paper by Koterski *et al* [40] on Venazuelan equine encephalitis virus (VEEV) is noteworthy.

IFN has been shown to have antiviral effect both against VSV [41] and CHPV [42] as evidenced by *in-vitro* and human studies respectively. Fig 10a clearly documents excessive upregulation of IFN gene in the early stages of the infection (24hr PI) followed by drastic decrease. Thus, though IFN is produced in large quantities to combat the infection, desired effect was not achieved leading to death. Importantly, no elevation was recorded in virus-infected P-2 siRNA treated as well as control mice receiving the vehicle alone. IL-10, involved in the suppression of the protective immune response was elevated in the initial phase and at the time of death. This cytokine might be playing crucial role in the pathogenesis of CHPV. The elevated expression of TNF and IL-1β were found to decrease with time that is consistent with a previous report documenting susceptibility of TNF-α and IL-1β knockout (KO) mice to lethal HSV-1 encephalitis [43]. SOCS-1, the suppressor of cytokine signaling gene playing a critical role in the negative regulation of cytokine signaling was steadily decreasing after initial elevation. These results are similar to a study with rabies virus infection wherein downregulation of SOCS-1 gene was shown [44].

The elevated levels of Ccl2 and Cxcl-10 suggest recruitment and activation of dendritic cells, macrophages, neutrophills and other white cells to the sites of infection. However, as TNF and IFN-γ are known to induce Cxcl-10, deceasing levels of TNF and IFN-γ correlate well with downregulation of Cxcl-10 in our study [45]. FasL and IL-2R were upregulated in the beginning of infection and deceased further. FasL is known to induce apoptosis in brain cells during viral encephalitis [46].

The gene profiling results clearly demonstrate that none of the genes modulated during the course of CHPV infection were affected in P-2 siRNA treated mice confirming that the siRNA did not allow replication of CHPV (real time and histopathology also scored the brains as negative for CHPV) and therefore did not lead to the cascade of immune reactions leading either to recovery or death. Absence of anti-CHP antibodies provides similar evidence.

In a nutshell, we report the identification of an efficient siRNA that can potently protect mice against lethal encephalitis induced by CHPV and utility of the cationic lipids for effective brain delivery. This preliminary but important finding should be taken to its logical conclusion by conducting additional studies mentioned earlier including trial in rhesus monkeys.
